# Parkinson's disease-related non-motor features as risk factors for post-operative delirium in spinal surgery

**DOI:** 10.1371/journal.pone.0195749

**Published:** 2018-04-09

**Authors:** Ki Hoon Kim, Suk Yun Kang, Dong Ah Shin, Seong Yi, Yoon Ha, Keung Nyun Kim, Young Ho Sohn, Phil Hyu Lee

**Affiliations:** 1 Department of Neurology, Yonsei University College of Medicine, Seoul, Republic of Korea; 2 Department of Neurology, Dongtan Sacred Heart Hospital, Hallym University College of Medicine, Hwaseong, Gyeonggi-do, Republic of Korea; 3 Department of Neurosurgery, Spine and Spinal Cord Institute, Yonsei University College of Medicine, Seoul, Republic of Korea; Oslo Universitetssykehus, NORWAY

## Abstract

**Background:**

The clinical features of postoperative delirium are similar to the core features of alpha synuclein-related cognitive disorders, such as Parkinson’s disease dementia (PDD) or dementia with Lewy bodies (DLB). Therefore, we hypothesized that the non-motor symptoms (NMSs) in Parkinson’s disease (PD), which precede the cardinal motor features of PD, are likely to be risk factors for developing postoperative delirium. We investigated the association between PD-related NMSs and postoperative delirium in old people undergoing elective spinal surgery.

**Methods:**

This study was a prospective study. Participants were aged 65 years and older and scheduled to undergo elective spinal surgery. During the enrollment period, 338 individuals were screened, 104 participants were included in the analysis. We assessed eight easily-assessed and representative PD-related NMSs 1 day before the scheduled surgery using tests or questionnaires for each symptom. The presence of delirium was determined by using the short version of the Confusion Assessment Method (CAM).

**Results:**

Fifteen (14.4%) of the 104 participants (age, 71.7 ± 4.7 years; men, 34.6%) met the CAM criteria for post-operative delirium. Multivariate logistic analysis showed that decreased olfactory function (odds ratio [OR] 0.63, 95% CI 0.44–0.91) and exhibiting rapid eye movement sleep behavior disorder (RBD, OR 1.45, 95% CI 1.09–1.93) were significantly independent predictors of postoperative delirium.

**Conclusions:**

Our study shows that hyposmia and RBD are significantly independent risk factors for postoperative delirium in general elderly population. Considering that NMSs may represent burden of alpha synuclein deposit, we postulate that an underlying alpha synucleinopathy may correlates with postoperative delirium.

**Significance:**

This study gives a novel insight for the risk factor of postoperative delirium.

## Introduction

Postoperative delirium is common in elderly patients, and is a clinical challenge for clinicians because of the close association of poor surgical outcomes and prolonged hospitalization [1, 2]. Many risk factors have been reported including age, cognitive decline, depression, medical comorbidity, psychotropic drug use, admittance to an intensive care unit, and type of surgical procedure, but the mechanism of the postoperative delirium is unclear.

Postoperative delirium has similarities to the core features of alpha synuclein-related cognitive disorders, such as Parkinson’s disease dementia (PDD) or dementia with Lewy bodies (DLB): fluctuating attention, visual hallucination, and disorganized thoughts [3, 4]. In terms of clinical similarity, delirium may be a series of these cognitive disorders [3, 5]. A common underlying neurochemical change supports their similarities. It is well known that decreased cholinergic levels that can be seen in the pathology of dementia with Lewy bodies play an important role in delirium [5]. Further, a previous study demonstrated that alpha synuclein pathologies are associated with postoperative delirium after gastrectomy [3]. Because of these clinical, and neuropathological similarities, we assumed that postoperative delirium could be a preclinical stage of alpha synucleinopathy.

Several non-motor symptoms (NMS) in Parkinson’s disease (PD), including rapid eye movement sleep behavior disorder (RBD), olfactory dysfunction, constipation, daytime sleepiness, insomnia, depression, anxiety, and orthostatic hypotension have been identified preceding cardinal motor features. Among these NMS in PD, olfactory dysfunction, constipation and RBD have been directly associated with abnormal synuclein pathologies [6, 7]. Although data are lacking on direct relationship between each other NMS and synuclein pathologies, NMS are considered as prodromal markers for PD which is representative alpha synucleinopathy [8].

We hypothesized that if general population without Parkinson`s disease have PD-related NMSs which implies underlying alpha synucleinopathy, they are likely to be vulnerable for post-operative delirium in elderly people undergoing spinal surgery.

## Materials and methods

### Participants

This study was a prospective study conducted in the neurology and neurosurgery departments of Yonsei University Severance Hospital (Registration: www.clinicaltrial.gov NCT 02550626). Participants were enrolled between October 2015 and July 2016. Eligible participants were age 65 years and older, scheduled to have elective spinal surgery at the neurosurgery department of Yonsei University Severance Hospital, and expected to be hospitalized for at least 3 days. Exclusion criteria included evidence of current or previous delirium before surgery, previous diagnosis of Parkinson’s disease, chemotherapy or radiotherapy within one year due to underlying malignancy, renal or hepatic insufficiency (estimated glomerular filtration rate < 50mL/min or pre-operative hepato-biliary department consultation due to liver enzyme elevation, respectively), severe quadriplegia patients who could not evaluate NMSs, and emergency surgery.

Eligible individuals who were scheduled to have spinal surgery the next day were assessed previous or current delirium using the Confusion Assessment Method (CAM) [9]. We used the CAM to exclude people with previous or current delirium [3, 10]. And then the Korean version of the Mini-Mental State Examination (K-MMSE) and neurologic examinations were assessed for the baseline evaluation. After baseline assessment, we checked PD-related NMSs. A neurologist trained for this purpose carried out the interviews and neurological examinations. Because various questionnaires and tests were required for evaluation, we confirmed that all participants were alert and able to communicate. A well-trained physician used the CAM to assess the patients for post-operative delirium on each of the three days after surgery, with the first assessment carried out within 24 hours of the operation. A neurologist also re-examined the studied population and reviewd their medical records. This protocol was approved by the ethics committee of Yonsei University Severance Hospital and written informed consent was obtained from all the participants.

All participants underwent general endotracheal anesthesia with propofol, remifentanil, and pancuronium or vecuronium as muscle relaxants and anesthesia was maintained with desflurane and nitrous oxide inhalation. Participants received vital sign monitoring during the operation, including radial arterial blood pressure and applied warming devices such as forced-air warming devices and warm circuit for maintaining body temperature. All patients received appropriate tracheal extubation before being transported to the ward or the intensive care unit. Surgical procedures in this study included cervical or lumbar decompression, fusion, and tumor removal.

### Assessment of Parkinson’s disease-related non-motor symptoms (NMS)

Parkinson’s disease-related NMSs were evaluated 1 day before scheduled surgery. We chose easily-assessed and representative eight NMSs: olfactory disturbance, constipation, orthostatic hypotension, insomnia, excessive daytime somnolence, rapid eye movement sleep behavior disorder (RBD), depression, and anxiety.[6–8]

#### Olfactory disturbance

The 12-item Cross-Cultural Smell Identification Test (CCSIT) was used to measure olfactory function [11]. We define normal olfactory function as 9 points or higher of the total of 12 points, and decreased olfactory function as 8 points or less, based on previous studies [11, 12].

#### Insomnia and daytime somnolence

We used the Insomnia Severity Index (ISI) to measure insomnia. The ISI is a brief screening instrument that measures sleep maintenance difficulties, satisfaction with current sleep patterns, interference with daily functioning, noticeability of quality of life, and the degree of distress or concern expressed by the participant about each item. The total score ranges from 0 to 28 and, based on previous validation research [13], a score of 8 or higher indicated the possibility of insomnia.

We employed the most frequently used method for measuring daytime sleepiness, the Epworth Sleepiness Scale (ESS) [14] to assess daytime somnolence. We defined excessive daytime sleepiness as an ESS score > 10, in line with previous Asian validation research [15].

#### Rapid eye movement sleep behavior disorder (RBD)

We used an easily-assessed questionnaire for screening RBD, a validated Korean version of the RBD screening questionnaire (RBDSQ-K) [16].We classified our patients as exhibiting RBD if their total RBDSQ-K score was 5 or higher, in line with an original study [17].

#### Depression

We assessed depression using the Korean version Beck Depression Inventory (BDI)[18, 19]. We diagnosed depression[20] in patients who scored ≥ 17 [20].

#### Anxiety

We assessed all patients against the Parkinson Anxiety Scale (PAS), a recently developed instrument that has been validated for PD patients [21]. This scale consists of a 12-item observer- or patient-rated scale and three subscales: persisting anxiety (five items), episodic anxiety (four items), and avoidance behavior (three items). We employed the same observer-rated cut-offs as in the original article for totals and subscales to diagnose anxiety disorders (generalized anxiety disorder, panic disorder, and avoidant anxiety disorder) [21]. We made a positive diagnosis in the following circumstances: PAS total score for generalized anxiety of 13 or higher; PAS subscale score for persistent anxiety of 9 or higher; PAS subscale score for episodic anxiety 3 or higher; and PAS subscale score for avoidance of 3 or higher.

#### Orthostatic hypotension

Participants undertook the orthostatic blood pressure test, except for 4 participants who were unable to take the test due to severe pain. After resting in supine position for least 5 minutes, a physician measured blood pressure and heart rate serially after change of posture from supine to standing. Blood pressure and heart rate were obtained in the supine position and immediately after standing, 2 minutes after standing, 5 minutes after standing. We classified our participants as exhibiting orthostatic hypotension if we confirmed a sustained blood pressure reduction (SBP ≥ 20 mmHg or DBP ≥ 10 mmHg) at any time within 5 minutes of standing. This is a modification of the traditional definition [22].

#### Constipation

Constipation is defined generally as fewer than 3 bowel movements per week [23, 24]. To avoid diagnostic complexity, we focused simply on the frequency of defecation and the use of laxatives. We classified as constipated participants with ≤ 2 bowel movements per week or taking laxatives for at least 3 months.

### Detecting post-operative delirium and neurological evaluation

The presence of delirium was assessed using the CAM.[9] Because the CAM is highly sensitive (sensitivity 94–100%), rapid, and simple, it is widely used for assessing delirium in high-risk settings. The CAM consists of 4 clinical features: acute onset (i.e change in mental status) and fluctuating course (feature 1), inattention (feature 2), disorganized thinking (feature 3), and altered level of consciousness (feature 4). A diagnosis of postoperative delirium requires the presence of features 1 and 2 and either feature 3 or 4. We ignored any symptoms or signs of delirium on the day of operation because of the immediate impacts of preoperative medication and anesthetic agents. Delirium assessments took place between 9am and 12 midday on days 1, 2, and 3 after surgery using the CAM diagnostic tool for interviews with participants and their caretakers. A trained neurologist identified the presence of delirium based on the CAM, and a second investigator validated any assessments of delirium. In the present study, we define postoperative delirium as the presence on any postoperative day of an acute confusional state that meets the CAM criteria.

### Statistical analysis

We compared participants with and without delirium across all the clinical data. The Pearson χ2 test or Fisher`s exact test for categorical variables was used to compare frequencies. For continuous variables, we examined the normality of their distribution using the Kolmogorov- Smirnov test. Provided the data did not deviate from a normal distribution, we calculated the mean and standard deviation and used independent sample t-tests for comparisons. In the case of data that were not normally distributed, we recorded the median and interquartile range (IQR) and compared them using the Mann-Whitney U test.

We performed logistic regression analysis to assess the association between postoperative delirium and outcome variables. For univariate logistic regression analysis, we compared post-operative delirium and possible confounding factors including age, sex, hypertension, diabetes, old cerebrovascular accident, cardiovascular comorbidity, previously diagnosed psychiatric or cognitive disorder, intensive care unit use after operation, perioperative factors such as blood loss and operation time, each PD-related NMS and prodromal symptoms scores. Variables with P<0.1 in the univariate analyses were entered into the multivariate model to identify independent predictors of postoperative delirium. The logistic regression analyses results are reported as crude and adjusted hazard ratios with 95% confidence interval (95% CI). SPSS for Windows (version 20.0; SPSS, Chicago, IL) was used as statistical software. *p* values <0.05 were regarded as significant.

## Results

### Participants

**Fig 1:**
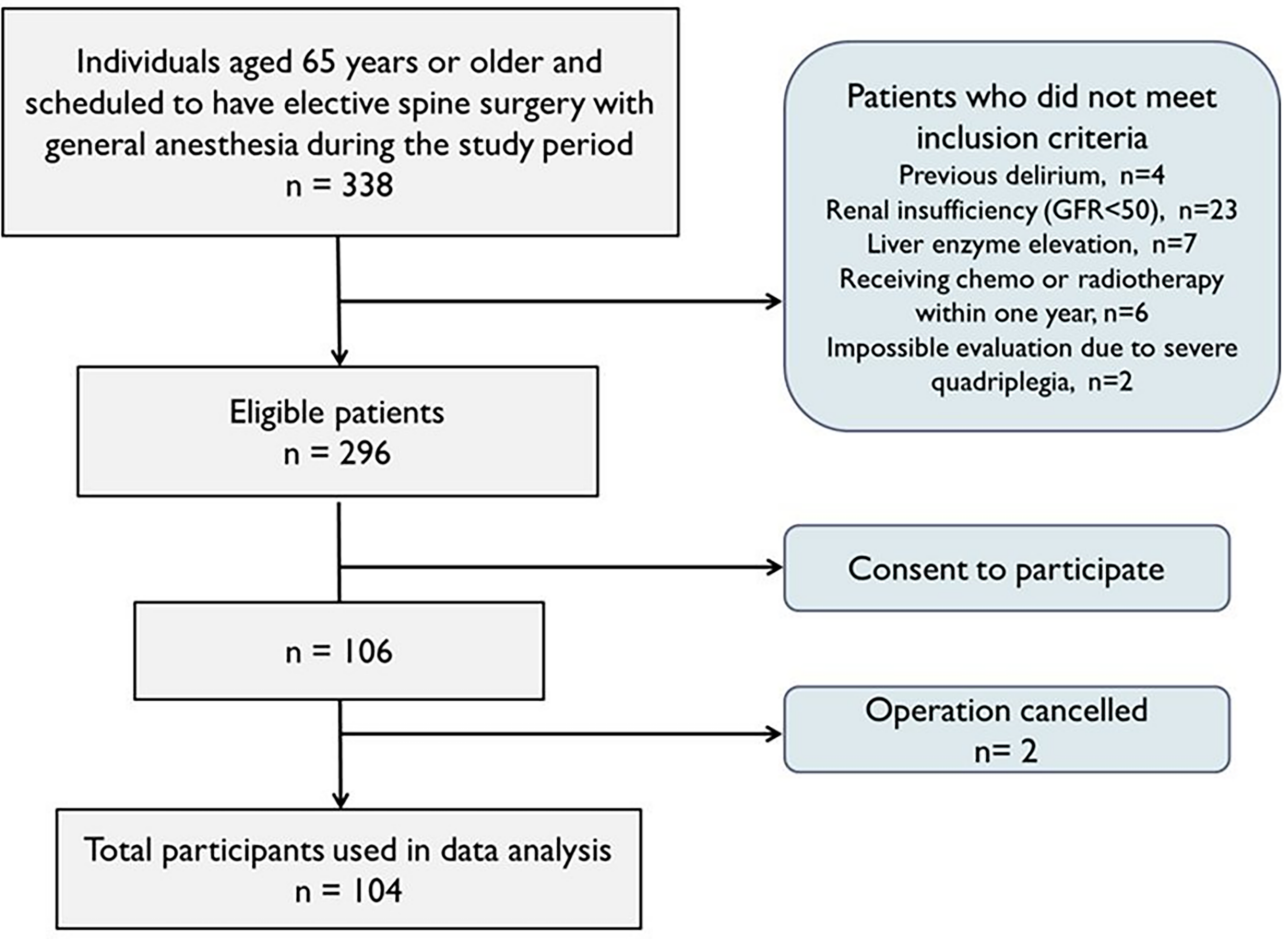
Patient enrollment. 104 participants of 296 eligible patients were included in the study analysis.

During the period of the study, 338 individuals were aged 65 years or older and scheduled to undergo elective spinal surgery at the neurosurgery department of our hospital. 106 (31.4%) patients agreed to participate. Therefore, 104 participants were included in the analysis because of cancellation of two patients (Fig 1). The characteristics and perioperative findings are summarized in Table 1. 13 of 104 participants were suspected of parkinsonism on neurologic examination, but additional tests for the differential diagnosis were not performed.

**Table 1:** Characteristics and peri-operative findings in participants.

		Total	Postoperative delirium		p-value
			Yes	No	
		(n = 104)	(n = 15)	(n = 89)	
Age (years), mean ± SD		71.7±4.7	73.2±4.7	71.5±4.6	0.185
Men		36 (34.6)	9 (60.0)	27 (30.3)	0.039
Education (years)		7.5 [6–12]	6 [6–12]	8 [6–12]	0.817
MMSE score (0–30)		27 [25–28]	25 [23–27]	27 [25–29]	0.023
Hypertension		67 (64.4)	10 (66.7)	57 (64.0)	0.844
Diabetes mellitus		24 (23.1)	5 (33.3)	19 (21.3)	0.308
Psychiatric disorder		13 (12.5)	3 (20.0)	10 (11.2)	0.395
Previous diagnosis of dementia or MCI		9 (8.7)	3 (20.0)	6 (6.7)	0.120
old CVA or TIA history		8 (7.7)	3 (20.0)	5 (5.6)	0.088
Cardiovascular comorbidity		23 (22.1)	5 (33.3)	18 (20.2)	0.258
Parkinsonism		13 (12.5)	5 (33.3)	8 (9.0)	0.008
Charlson Comorbidity Index		3 [2–4]	3.4 [3–5]	3 [2–4]	0.353
Number of drugs before operation		5 [3–7]	5 [2–7]	5[3–7]	0.755
	Statin	50 (48.1)	6 (40.0)	44 (49.4)	0.499
	ARB	39 (37.5)	5 (33.3)	34 (38.2)	0.719
	Anti-thrombotics	30 (28.8)	5 (33.3)	25 (28.1)	0.678
	Psychoactive drugs^a^	33 (31.7)	6 (40.0)	27 (30.3)	0.457
Preoperative laboratory findings					
	Albumine (g/dL)	4.12 ± 0.33	4.07 ± 0.30	4.12 ± 0.33	0.595
	BUN (mg/dL)	16.9 ± 4.96	15.7 ± 4.50	17.1 ± 5.03	0.331
	Creatinine (mg/dL)	0.77 ± 0.20	0.81 ± 0.19	0.76 ± 0.20	0.343
	Hemoglobin (g/dL)	13.6 ± 1.38	13.5 ± 1.25	13.6 ± 1.40	0.809
	WBC (10^3/μL)	7.064 ± 1.71	6.834 ± 1.15	7.103 ± 1.79	0.452
	Platelet (10^3/μL)	232 ± 57.1	230 ± 47.7	233 ± 58.8	0.840
Surgical site					0.319
	Cervical	19 (18.3)	2 (10.5)	17 (19.1)	
	Thoracic	2 (1.9)	1 (5.3)	1 (1.1)	
	Lumbar	83 (79.8)	12 (63.2)	71 (79.8)	
Surgical method					0.729
	Spinal fusion	77 (74.0)	12 (80.0)	65 (73.0)	
	Decompression	22 (21.2)	2 (13.3)	20 (22.5)	
Admission to ICU		13 (12.5)	3 (20.0)	10 (11.2)	0.395
Blood loss during operation (cc)		350 [200–937]	250 [100–800]	400 [200–975]	0.127
Operation time (min)		183 [142–234]	157 [120–202]	184 [142–239]	0.197
Presence of postoperative fever (>37.8)		50 (48.1)	7 (46.7)	43 (48.3)	0.906

Abbreviations: SD = standard deviation; MCI = mild cognitive impairment; CVA = cerebrovascular accident; TIA = transient ischemic attack; ARB = angiotensin II receptor blocker; ICU = intensive care unit

Data are expressed as mean ± SD, median [interquartile range] or number (%).

^a^Psychoactive drugs included prescribed anti-psychotics, sedative hypnotics, benzodiazepins, opioids, anti-histamine, anti-cholinergics, and dopaminergic medications.

### Comparison of Parkinson’s disease-related NMSs between two groups with and without postoperative delirium

CCSIT and RBDSQ-K scores were significantly different between the 2 groups (*ps*<0.001, respectively), while other variables were similar. Hyposmia and RBD, determined by CCSIT and RBDSQ-K scores, were significantly associated with postoperative delirium (relative risk 1.33, 3.63, respectively; Table 2).

**Table 2:** Comparison of Parkinson’s disease-related NMSs between subjects with and without post-operative delirium measured by each instrument.

	Postoperative delirium		p-value	Relative risk (95% CI)
	Yes	No		
Test	(n = 15)	(n = 89)		
Hyposmia (CCSIT score < 9)	12 (80.0)	30 (33.7)	0.001	1.33 (1.09, 1.63)
Daytime sleepiness (ESS >10)	1 (6.7)	2 (2.2)	0.376	
Insomnia (ISI >7)	5 (33.3)	22 (24.7)	0.481	
RBD (RBDSQ-K >4)	6 (40.0)	2 (2.2)	<0.001	3.63 (1.09, 12.06)
Constipation	3 (15.8)	16 (18.0)	1.000	
Any anxiety disorder	6 (40.0)	21 (23.6)	0.180	
Orthostatic hypotension	1 (6.7)	14 (16.1)	0.685	
Depression (BDI > 16)	7 (50.0)	27 (31.4)	0.173	

Abbreviations: CCSIT = Cross-Cultural Smell Identification Test; ESS = Epworth Sleepiness Scale; ISI = Insomnia Severity Scale; RBDSQ-K = Korean version of RBD screening questionnaire; OH = Orthostatic Hypotension; BDI = Beck Depression Inventory

Data are expressed as the median [interquartile range] or number (%).

### Univariate and multivariate logistic regression analyses for predicting postoperative delirium

When we analyze various confounding factors affecting delirium using univariate logistic regression, postoperative delirium occurred more frequently occurred in men and those with low CCSIT or high RBDSQ-K scores. In multivariate analyses using variables with *p*-value <0.1 in the univariate analyses, CCSIT and RBDSQ-K scores (*p<*0.010, *p<*0.005 respectively) were independently associated with postoperative delirium. Elderly patients with low CCSIT or high RBDSQ-K scores had a greater possibility of postoperative delirium after spinal surgery (Table 3).

**Table 3:** Univariate and multivariate logistic analyses for factors associated with postoperative delirium.

	Univariate		Multivariate^a^	
	OR (95% CI)	p value	OR (95% CI)	p value
Age	1.08 (0.96–1.22)	0.187		
Men	3.44 (1.12–10.64)	0.032	1.16 (0.22–6.17)	0.860
Hypertension	1.12 (0.35–3.57)	0.845		
Diabetes mellitus	1.84 (0.56–6.04)	0.313		
Cardiovascular comorbidity	1.97 (0.60–6.49)	0.264		
Psychiatric disease	1.98 (0.48–8.22)	0.35		
Previous dementia or MCI	3.46 (0.76–15.69)	0.108		
Previous old CVA or TIA	4.20 (0.89–19.87)	0.070	1.99 (0.20–19.82)	0.560
MMSE score	0.85 (0.71–1.03)	0.094	0.86 (0.66–1.12)	0.262
admission to ICU	1.98 (0.48–8.22)	0.350		
Blood loss during operation (cc)	0.999 (0.998–1.001)	0.234		
Operation time (min)	0.994 (0.986–1.002)	0.154		
Parkinsonism	5.06 (1.39–18.51)	0.014	1.63 (0.26–10.34)	0.606
Parkinson`s disease-related NMS				
CCSIT score	0.57 (0.42–0.76)	<0.001	0.63 (0.44–0.91)	0.014
ESS score	1.06 (0.88–1.27)	0.534		
ISI score	1.01 (0.91–1.12)	0.898		
RBDSQ-K score	1.66 (1.27–2.16)	<0.001	1.45 (1.09–1.93)	0.010
BDI score	1.05 (0.99–1.11)	0.131		
Constipation	1.14 (0.29–4.52)	0.851		
Anxiety disorder	2.16 (0.69–6.77)	0.187		
Orthostatic hypotension	0.57 (0.12–2.73)	0.479		

Abbreviations: OR = odds ratio; CI = confidence interval

^a^Variables which showed P<0.1 in the univariate analysis were included in the multivariate analysis.

## Discussion

Our study shows olfactory dysfunction and RBD are significantly independent risk factors for the postoperative delirium. This suggests that PD-related NMSs may contribute to the development of postoperative delirium, and implies that alpha synucleinopathy may predispose to postoperative delirium.

Previous studies about postoperative delirium report many risk factors [2]. In our bivariate analysis, gender, K-MMSE scores, and parkinsonism were significantly different between the with-delirium and without-delirium groups (p = 0.039, p = 0.023, p = 0.008, respectively). Cognitive impairment and low MMSE scores are well known as predisposing factors for postoperative delirium [25]. Further, this study suggests that men are at higher risk of postoperative delirium after spinal surgery. Several studies suggest that sex differences affect the likelihood of developing postoperative delirium, although there are many conflicting views [26]. These differences might be due to differences between the immune systems of men and women [27]. Very few studies were concerned about increased risk of post-operative delirium in PD [28], and decreased cerebral acetylcholinergic activity is presumed to be its mechanism [3]. However, in case of parkinsonism, discussion may be limited in our study, because there are many etiologies of parkinsonism and no further investigation for the diagnosis were conducted.

However, age, medical comorbidities, number of used drugs before operation, and psychotropic drug use, which are well-known predisposing factors [2], did not differ significantly between the two groups. The proportion of patients having preoperative old CVA or TIA was higher for those with postoperative delirium than those without, although this trend was not statistically significant (*p* = 0.088). An earlier analysis of a large retrospective database, lumbar fusion was associated with a greater risk of postoperative delirium than lumbar decompression [29], but our results did not show an association between surgical method and postoperative delirium. These statistical uncertainties about previously known factors may be explained by the relatively small number of participants and relatively homogenous group in present study.

Decreased olfactory function is strongly associated with postoperative delirium. Because olfactory dysfunction precedes motor symptoms by several years, and affects up to 90% of PD patients over time; it is considered a preclinical marker of PD [6]. Olfactory dysfunction in PD is suggested to be due to alpha synucleinopathy [8]. Given the association between hyposmia and postoperative delirium, we postulate that postoperative delirium might be the preclinical stage of alpha synucleinopathy. A recent study has shown similar results that preexisting decreased olfactory function is associated with postoperative delirium in cardiac surgery [30].

Our study reveals that RBD is a strong independent risk factor for postoperative delirium. Previous cohort studies demonstrated that idiopathic RBD patients has high risk of PD and related alpha-synucleinopathy [31]. Further, postmortem pathologies in idiopathic RBD patients have identified widespread Lewy bodies pathology [32]. This strongly suggests that RBD may be the prodromal stage of alpha synucleinopathy-related neurodegenerative diseases. Our result supports the association between alpha synuclein pathologies and postoperative delirium.

We classified the eight (7.7%) participants in the study who obtained a score of 5 or higher for the RBDSQ-K as exhibiting RBD. There may be an argument that this is a greater rate than previous studies of the general population [33, 34]. However, these previous studies have also some limitations: possible selection bias because of survey of co-occurrence with RBD and sleep-related injury [33] and telephone interview without polysomnography (PSG) [34]. A recent study of the Korean elderly population aged 60 years or above has suggested that the prevalence of RBD and subclinical RBD confirmed by PSG is approximately 2% and 5% respectively [35]. This is similar to our results, once subclinical RBD is included. Therefore, the proportion of RBD patients assessed by RBDSQ-K in our study was reasonable.

The evidence of prodromal NMSs in PD is based on extranigral alpha-synuclein deposition patterns [36]. The olfactory bulb and lower brain stem involvement before substantia nigra pars compacta may explain NMS. An earlier study using the NMSQuest questionnaire showed that the number of NMSs correlates with disease duration and severity in PD [6]. Thus, in pathological and clinical aspects, NMSs may be useful clinical markers for detecting alpha synucleinopathy and the combination of NMSs may open the possibility of predicting PD in the future [37, 38]. Therefore, we assume that detecting NMSs in general population without Parkinson`s disease may reflect the underlying burden of alpha synuclein deposit. In our study, decreased olfactory function and exhibiting RBD, which are representative NMS in PD, correlate with a greater risk of postoperative delirium. With further investigation, measuring and detecting these two NMSs may be useful as predictive factors for postoperative delirium to assist surgeons in making a decision about undertaking high-risk surgery in a planned and non-emergency surgical procedure.

This is the first reported prospective study to analysis the correlation between PD-related NMSs and postoperative delirium in general population. The major finding of our study is the significant correlation between PD-related NMSs and postoperative delirium. Among PD-related NMS, RBD and olfactory dysfunction were independent risk factors for predicting postoperative delirium. This finding suggests that postoperative delirium may be a preclinical stage of alpha synucleinopathy. We suggests that RBD and hyposmia should be added in the list of predictors for postoperative delirium.

Our study has some limitations. First, not all eligible participants agreed to take part in this study. Only one third of all eligible people were enrolled. Evaluating PD-related NMSs took more an hour, and many people were reluctant to participate to this study the one day before the operation. The number showing post-operative delirium was small. In this study, several well-known predisposing factors for postoperative delirium did not produce statistically significant results may be due to the limited number of participants. A larger sample is needed to solidify the correlations between non-motor features and postoperative delirium. Second, we used indirect questionnaires to measure non-motor features rather than specific diagnostic methods, for example, PSG. NMS assessments using these questionnaires are dependent on subjects’ recall, which may be affected by cognitive function or reporting bias. Third, when designing this study, we had assumed that postoperative delirium might be a preclinical phase of alpha synucleinopathy from their clinical and neurochemical similarities. Long-term follow-up data must be available to confirm this assumption. Besides, more clinical information (i.e., response to treatment of delirium, the outcome, and the long-term follow-up) may be needed to demonstrate the clinical implications of our findings. Fourth, more information of spine specific variables (surgical procedure, primary or revision, and number of operative levels) would provide additional utility of our study and contribute further to the spine literature.
